# Artificial Intelligence Enhanced Virtual Reality: A Personalized, Multilingual Approach to Pain Management

**DOI:** 10.1089/jmxr.2024.0038

**Published:** 2024-11-26

**Authors:** Oranicha Jumreornvong, Hanchen Mao, Gonzalo Povea Galdo, Taraf Jaro, Kaitlyn Schuler, Christopher L. Robinson, Qing Zhao Ruan

**Affiliations:** 1 Department of Human Performance and Rehabilitation, Icahn School of Medicine at Mount Sinai, New York, New York, USA.; 2 High School of Health Profession and Human Services, New York, New York, USA.; 3 Department of Biomedical Engineering, Universidad Peruana Cayetano Heredia, San Martin de Porres, Peru.; 4 Royal College of Surgeons in Ireland Medical University in Bahrain, Busaiteen, Bahrain.; 5 Department of Psychology, University of North Carolina Wilmington, Wilmington, North Carolina, USA.; 6 Department of Anesthesiology, Perioperative, and Pain Medicine, Harvard Medical School, Brigham and Women’s Hospital, Boston, Massachusetts, USA.; 7 United States Department of Veterans Affair, The Warren Alpert Medical School of Brown University, Providence, Rhode Island, USA.

**Keywords:** artificial intelligence, virtual reality, chronic pain, pain psychology, diversity

## Abstract

In this commentary, we introduce the artificial intelligence (AI) integration of generative virtual reality (VR) and multilingual voice cloning as a novel approach to personalized pain management. Globally, pain impacts billions of people daily. AI generated VR technology has the potential to modulate pain and enhance cognitive engagement through rapid design and personalization, and AI voice cloning uses familiar, multilingual voices to offer both neurological and psychological support. Through demonstration of our prototypes’ videos, we aim to explain how a combination of personalized AI text to 3-dimensional generated VR landscape and AI cloned voices could potentially reduce pain by providing personalized, emotionally supportive treatment that targets psychological and emotional factors in chronic pain. Our next steps include conducting feasibility and clinical trials to validate the technology’s effectiveness across various cultural and health care settings.

## Introduction

The International Association for the Study of Pain defines pain as “an unpleasant sensory and emotional experience associated with actual or potential tissue damage or described in terms of such damage.”^1^ Pain affects billions of people worldwide, impacting both physical and psychosocial aspects of health.^2^ Chronic pain, which lasts greater than 3 months, is a complex condition involving interplay of the peripheral and central nervous systems. Common symptoms include anxiety, depression, social isolation, and reduced quality of life, often leading to functional impairment and financial burden.^3^

The biopsychosocial model of pain emphasizes chronic pain resulting from the complex interaction of biological, psychological, and social factors, including previous trauma. Psychiatric sequelae of chronic pain, such as addiction, overdose, chronic suicidal ideation, and suicide mortality, often arise due to the ongoing physical suffering and emotional distress experienced by patients.^4^ Consequently, adequate chronic pain management requires comprehensive, resource-intensive approaches addressing physical, psychological, and emotional aspects. In the 1990s and early 2000s, opioid prescriptions surged, significantly impacting pain treatment. However, the negative consequences of overuse eventually yielded a decline in opioid prescriptions, sparking innovation and paving the way for novel, targeted pain treatments.^5^

Artificial intelligence (AI) may improve pain management by providing personalized care and improving clinical decision making. AI has been incorporated into diagnostics, predicting pain progression, treatment response, and therapeutic management performance.^6^ Virtual reality (VR) has also been shown to effectively reduce pain intensity in a range of acute pain situations, including procedural pain, as well as in chronic pain conditions via mechanisms like distraction from pain, anxiety reduction, and cognitive engagement. Using conversational avatars and immersion, VR offers further pain assessment and symptom collection advantages.^7^ In light of these advantages, personalized AI/VR environments through AI text to 3-dimensional (3D) content creations and AI voice cloning of loved ones could enhance the therapy effectiveness through cognitive distraction, visuotactile stimulation, visuomotor stimulation, and emotional support. Therefore, patients could rapidly customize their environment settings and audio based on their preferences.

## The Role of AI Voice Cloning in Personalized Care

AI voice cloning technology, such as Tacotron 2 or WaveNet, uses deep learning algorithms to replicate voices with high accuracy by determining the ratio of correctly aligned input characters using the attention matrix. This method replicates words and captures the unique pitch, tone, and rhythm of a person’s speech. Incorporating cloned voices from significant individuals, whether living or deceased, creates a more personalized and human-like audio experience, potentially influencing oxytocin pathways to alleviate pain and anxiety while enhancing its analgesic effect in chronic pain patients. Evidence indicates that a familiar voice, particularly one from a loved one, can activate cerebral regions associated with social safety and emotional regulation, such as the auditory cortex, amygdala, medial prefrontal cortex, and posterior cingulate cortex. This network’s activation extends to the periaqueductal gray, a crucial area for pain modulation, which suggests that familiar voices can significantly impact pain perception and provide relief in a way that feels emotionally supportive and safe.^8^ Moreover, language processing itself plays a critical role in pain perception, especially for chronic pain patients, where the nuances of familiar speech or comforting language can modulate pain experience. Research demonstrates that incorporating a patient’s native language and voice tone fosters these neural pathways, potentially enhancing the effectiveness of therapeutic interventions.^9^ Incorporation of AI voice cloning in over 20 languages expands the intervention’s reach, engaging these pathways in non-English-speaking patients and further tailoring pain management.

**FIG. 1.**
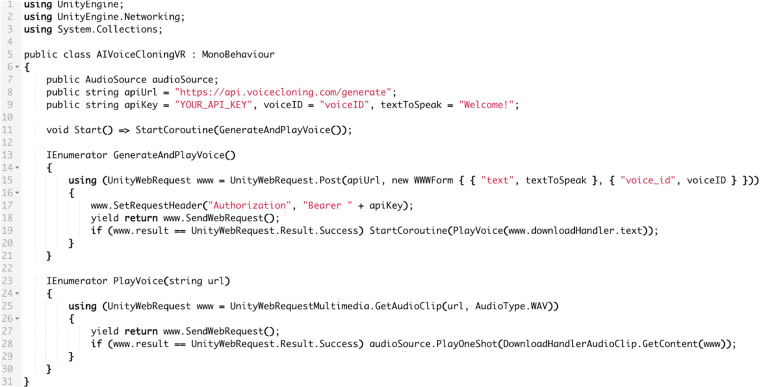
Simplified Code Sample for AI Voice Cloning and VR integration AI, artificial intelligence; VR, virtual reality.

The technology implemented for the study enhances pain management by using personalized, AI-generated voices that replicate familiar individuals with ElevenLabs’ voice cloning, leveraging Tacotron and WaveNet for high-quality, lifelike speech synthesis seen in figure 1. Please see in supplemental media 1 and 2 for the original recordings in Mandarin and English prior to AI cloning. These personalized AI cloned voices engage neural pathways linked to emotional processing and pain modulation, creating a calming and supportive environment across diverse linguistic backgrounds. While it may incorporate some principles of emotional processing during acute pain symptoms, its primary focus is on creating a personalized and calming environment through VR, rather than engaging in cognitive restructuring typical of cognitive based therapy (CBT), a psychological treatment that helps individuals reframe negative thought patterns. The AI cloned voice narration is not specifically trained to deliver CBT; instead, it guides patients through the VR environment, supporting emotional regulation and relaxation in ways that align with some aspects of CBT without being CBT itself. Nevertheless, AI offers the potential to transform CBT products by enabling personalized, real-time support, automated progress tracking, and data-driven insights, making therapy more accessible and effective for diverse users.

It is important to note that incorporating cloned voices from both living and deceased individuals raises substantial ethical considerations. These include the rights individuals hold over their likenesses and the necessity for protective measures to ensure that this technology is used responsibly, particularly in sensitive contexts like health care. The IEEE’s report, *Who Owns Our Second Lives: Virtual Clones and the Right to Your Identity*, delves into these issues, highlighting the importance of establishing guidelines and safeguards around AI-generated likenesses and virtual clones to protect both patients and voice contributors from potential misuse.^10^ Therefore, while AI voice cloning presents new opportunities in health care, especially for pain and anxiety management, it is essential to prioritize ethical frameworks and identity rights to ensure the protection of all involved.

## Prototype Development: AI Voice Cloning and AI Generative VR

The prototype leverages Figmin XR’s software to create personalized, interactive virtual reality environments designed to help patients manage chronic pain. Chronic pain is not solely a physical sensation. It has deep psychological roots, often exacerbated by feelings of isolation, anxiety, and stress. Figmin XR’s gamified features enable patients to construct their own virtual worlds that align with their personal preferences, fostering a sense of control and empowerment, which can positively influence their pain perception.

On the contrary, integrating AI voice cloning APIs within Unity’s VR space enhances personalized virtual environments by adding cultural and emotional relevance. This technology replicates familiar voices, including those of family members, living or deceased, tapping into neurological and psychological mechanisms of pain processing. Multilingual capabilities allow users to customize voices based on personal or cultural preferences, creating an immersive experience guided by loved ones. The system has the potential to trigger personalized voice responses based on VR interactions, offering a comforting, patient-centered approach to pain management that shifts focus away from pain and improves overall well-being.

**FIG. 2.**
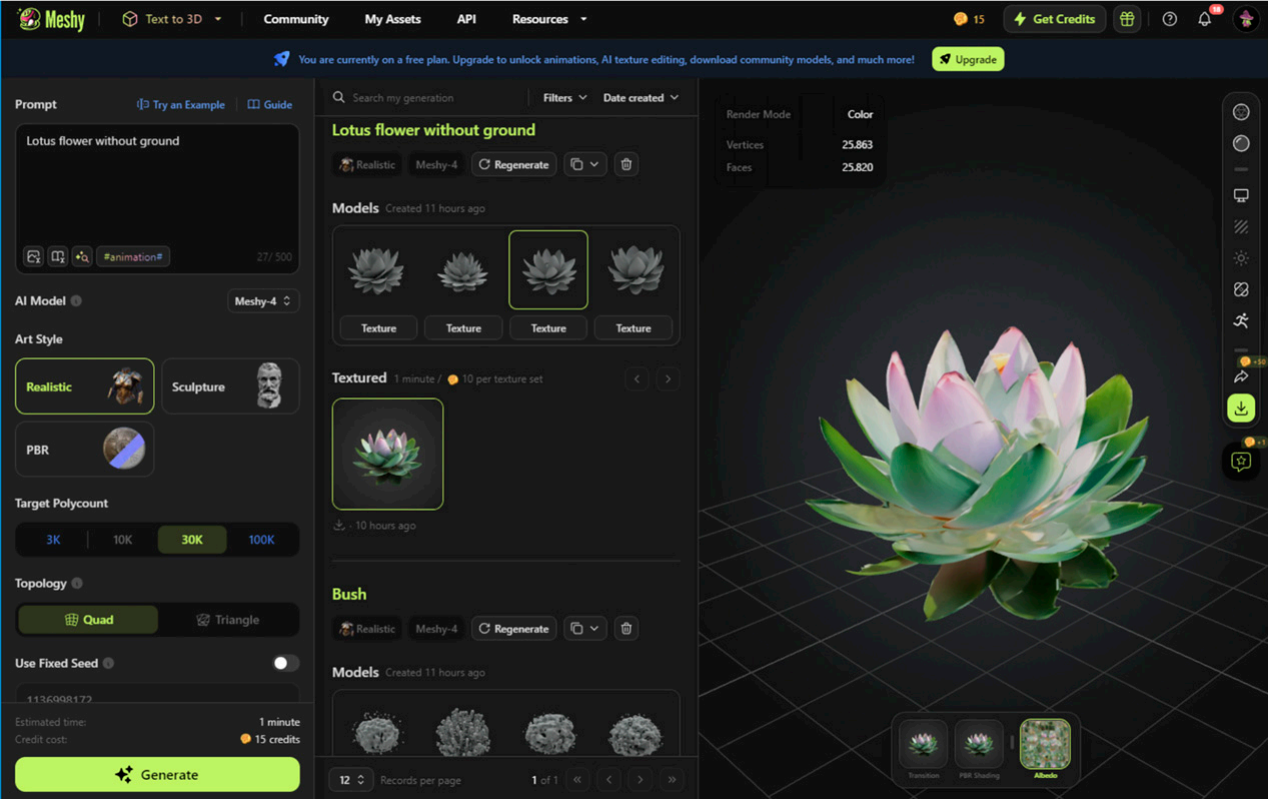
AI generative text to 3D shapes. AI, artificial intelligence.

Our second prototype was created in Unity and enhanced using various AI-based technologies. The concept design was initially generated using DALL-E using the text prompt: “Generate a relaxing island setting. This should contain elements such as flowers, trees, animals, and other details. The image’s purpose is to be relaxing, and the island must be seen as realistic as possible.” This AI-driven image generation tool creates detailed visual concepts based on text prompts, helping us visualize a relaxing island setting. Key elements such as palm trees, flowers, and stems were generated using the “Meshy AI 3D” generator, an AI tool for creating basic 3D models seen in figure 2. However, Meshy AI 3D struggled with intricate details, such as bushes and grass, at this time due to its current level of development. To address these limitations, we used “Sloyd,” a platform that utilizes a preexisting 3D model library where a model is introduced, and AI is utilized to modify the model. Sloyd’s distinct workflow allows for customizable and complex models, including plants and landscapes, enhancing our ability to create a naturalistic environment. The scene’s terrain was then built and textured using Unity Muse Texture, part of Unity’s AI suite for generating realistic environmental textures like sand and grass. In addition, Unity Muse Sprite, another feature in Unity’s AI toolkit, was utilized to generate a sun sprite for a sunset effect. This AI/VR technology can be tailored for therapeutic applications by creating personalized settings that help distract patients, reduce anxiety, and potentially enhance cognitive-behavioral interventions through real-time adjustments to match patients’ pain levels.

## Future Directions: Scaling and International Studies

Potential strengths of AI integration include its multilingual capabilities, being able to clone voice records of loved ones in 20+ languages, and ability to customize VR environments rapidly through generative text to 3D content. Real-time adjustments may be handled by the participant asynchronously through AI prompts, allowing for more intuitive, user-driven modifications in the VR environment without requiring direct intervention from an operator. This setup facilitates a seamless, adaptive experience for the user, relying on AI to adjust interactions based on input rather than continuous manual oversight. The text-to-3D animated scenes utilize generative AI to create fully realized 3D environments within Unity. These scenes are not only populated with static 3D models but also include dynamic, animated elements that can adapt to user interactions, providing a more immersive and responsive experience. This versatility could enhance global accessibility and patient engagement, allowing it to be applied in culturally and linguistically diverse populations. By creating customizable visual and voice interaction with patients, the technology has the potential to personalize care.

As emphasized earlier, the application of AI voice cloning, particularly for individuals who have passed, raises significant ethical considerations. To ensure respect for patient autonomy and likeness, explicit consent protocols must be established, addressing ownership of voice and likeness, particularly for voice records cloned from deceased individuals. Integrating clear consent measures will help safeguard patient and family rights, promoting trust in the ethical deployment of AI-driven tools.

The next step is to conduct a feasibility study using a mixed methods approach to evaluate a personalized AI/VR pain management device. Surveys will be distributed to three key stakeholder groups: health care providers, patients, and caregivers. The study aims to evaluate the perceived usefulness, interest, and potential barriers to implementing this AI/VR technology for chronic pain management across inpatient and outpatient settings, for both cancer-related and non-cancer chronic pain conditions, focusing on its international application and how different stakeholders, including patients, clinicians, and administrators, perceive its effectiveness, severity, access, and cultural acceptance. The findings will help refine our product by identifying user preferences for features such as AI voice cloning, multilingual support, and generative AI for text-to-3D animated scenes.

In addition, Institutional Review Board approval is sought to conduct clinical trials to evaluate the effectiveness of this technology in both high- and low-resource settings. Given the diversity of health care environments, these trials will assess cost-effectiveness, accessibility, and user experience across various health care systems. In low-resource settings, 3 Degrees of Freedom (3DOF) viewers and smartphones may be the primary hardware for deployment. While not excluding 6DOF mobile VR headsets in high-resource settings, this approach emphasizes the adaptability of the software for lower-end hardware to maximize accessibility. These trials will examine its cost-effectiveness and accessibility across different health care systems, with a focus on regions where resources are limited. Next steps also include collaborating with physicians, computer scientists, and stakeholders in multiple countries to refine and tailor this technology appropriate to the patients’ cultural and institutional settings. These partnerships will be crucial for evaluating this technology and gradually adapting it to unique needs in various health care conditions. This can also be done by working closely with hospitals, rehabilitation centers, and home care providers, to customize the technology to optimize its effectiveness in various environments. Moreover, these applications will help identify more applications for this technology, ensuring its optimal benefits for diverse patients.

## Conclusion

In conclusion, integrating immersive VR environments with AI voice cloning and AI generative technology contain a promising and transformative approach to pain management, including physical and psychological dimensions of chronic pain. This technology could not only provide a way to reduce pain perception but also could provide psychological support by creating a more personalized health care experience. Feasibility studies and clinical trials would be used to validate the effectiveness of this technology, with the global adoption for improving patient care in pain management. As the technology continues to develop, it could offer a scalable, personalized, and emotionally supportive pain management tool that can be applied worldwide.

## Abbreviations Used

3D: 3-dimensional
AI: Artificial Intelligence
CBT: Cognitive based therapy
DOF: Degrees of Freedom
VR: Virtual Reality
